# New molecular mechanisms of quercetin in improving recurrent spontaneous abortion based on in-depth network pharmacology and molecular docking

**DOI:** 10.3389/fchem.2024.1407667

**Published:** 2024-09-04

**Authors:** Dan Wang, Xuebing Li, Yifan Li, Ruilin Wang, Chunxia Wang, Yongwei Li

**Affiliations:** ^1^ The Second Clinical Medical College of Henan University of Chinese Medicine, Zhengzhou, China; ^2^ Henan Province Hospital of Traditional Chinese Medicine, The Second Affiliated Hospital of Henan University of Chinese Medicine, Zhengzhou, China

**Keywords:** network pharmacology, molecular docking, quercetin, recurrent spontaneous abortion, HTR-8/SVneo cells

## Abstract

**Introduction:**

The increasing prevalence of recurrent spontaneous abortion (RSA) poses significant physical and psychological challenges for affected individuals. Quercetin, a natural plant flavonoid, shows promise in reducing miscarriage rates, yet its precise mechanism remains elusive. This study uses network pharmacology, molecular docking, and experimental validation to explore the molecular pathways through which quercetin mitigates RSA.

**Methods:**

Quercetin-related target genes were sourced from the Traditional Chinese Medicine Systems Pharmacology Database and Analysis Platform (TCMSP), and RSA target genes were retrieved from the Comparative Toxicogenomics Database (CTD), with overlapping targets identified using Venn diagrams. All genes were visualized using the STRING database, and core targets were selected with Cytoscape 3.7.3. Gene ontology and Kyoto Encyclopedia of Genes and Genomes (KEGG) pathway enrichment analyses were conducted using the DAVID and Reactome online resources. Subsequently, HTR-8/SVneo cells were stimulated with lipopolysaccharide (LPS) and treated with varying concentrations of quercetin (1, 5, and 10 μM), then subjected to CCK-8, wound healing, transwell, and annexin V-FITC/PI apoptosis assays. Reverse-transcription quantitative PCR was used to determine the mRNA expression levels of IL-1β, TNF-α, and IL-6 in LPS-induced cells post-quercetin intervention, and western blotting was used to measure AKT1, MMP9, and caspase-3 protein levels.

**Results:**

A total of 139 quercetin-associated target genes were identified from the TCMSP database, and 98 disease-associated target genes were obtained from the CTD, resulting in 25 shared target genes. Gene ontology enrichment highlighted the involvement of these targets in positive regulation of apoptosis, response to hypoxia, and intrinsic apoptotic signaling pathway in response to DNA damage. KEGG pathway analysis indicated enrichment in pathways related to interleukin-4 and interleukin-13 signaling, cytokine signaling in the immune system, and apoptosis. Molecular docking studies revealed robust binding of quercetin with MMP9, AKT1, IL-1β, TNF, and caspase-3. *In vitro* experiments demonstrated that quercetin enhanced LPS-induced cell activity, fostering proliferation, migration, and invasion, and reducing apoptosis. Moreover, quercetin reduced IL-1β, TNF-α, and IL-6 mRNA expression, increased AKT1 and MMP9 protein levels, and reduced caspase-3 expression.

**Conclusion:**

Quercetin could mitigate the incidence of RSA by modulating inflammatory responses and apoptotic processes, through upregulation of AKT1 and MMP9, and downregulation of caspase-3, IL-1β, TNF-α, and IL-6. Quercetin opens up a new way of thinking about treating RSA.

## 1 Introduction

Recurrent spontaneous abortion (RSA), which is clinically defined as the occurrence of three or more consecutive spontaneous abortions before the 24th week of gestation in women with consistent sexual partners, affects approximately 1%–5% of women of reproductive age (Tomkiewicz and Darmochwał-Kolarz, 2023). Its etiology is complex and includes genetic factors, immunological factors, endocrine disorders, reproductive dysfunction, and infections (Garrido-Gimenez and Alijotas-Reig, 2015; Hong Li and Marren, 2018; Deng et al., 2022; Turesheva et al., 2023). The main clinical strategies for treatment of RSA are based on immunotherapy, anticoagulant therapy, hormonal supplementation, and micronutrient supplementation (Saccone et al., 2017; Awolumate et al., 2020; Zhou H. et al., 2022). However, the efficacy of these strategies remains limited, and they have the potential to cause adverse effects. Repeated miscarriages can have severe physical and psychological repercussions for RSA patients, potentially extending to family and society. Therefore, there is an urgent need to find more effective treatments for RSA.

Traditional Chinese Medicine (TCM) has been used for more than 3,000 years to treat common pregnancy problems (Li et al., 2016). Previous studies have shown that TCM can have a complementary role to Western medicine, in that it can significantly reduce toxicity and side-effects, improve efficacy, prevent abortion, and promote pregnancy (Lai et al., 2010; Li H. et al., 2020; Song et al., 2023). Herbal medicines are particularly important in the treatment of RSA. Therefore, highly effective herbal medicines with low-toxicity have good application prospects.

Quercetin is a natural plant-derived flavonoid polyphenol that is widely found in edible plants such as vegetables and fruits. In addition, quercetin is found in some commonly used kidney tonic and fetus-restoring Chinese medicines such as Semen Cuscutae and Herba Taxilli (Wu et al., 2024). Many studies have shown that it has a wide range of pharmacological effects, exerting anti-inflammatory, antibacterial, antioxidant, anti-tumor, and anti-viral effects, as well as other biological activities, and that it has an important role in various diseases (Carrillo-Martinez et al., 2024). Its ability to modulate immune responses at the maternal–fetal interface enables it to influence extrachorionic trophoblastic cell migration and invasion, potentially ameliorating adverse pregnancy outcomes (Zhou J. et al., 2022; Fu et al., 2022). However, the precise mechanism through which quercetin mitigates miscarriage requires further elucidation.

Network pharmacology is a comprehensive methodology for identifying disease–drug–target relationships, facilitating an in-depth understanding of drug pharmacology and biological network effects. A study based on network pharmacology previously found that quercetin has an important role in the traditional Chinese quadri-combination formula [Bushen, Yiqi, Lixue, and Yangtai (BYLY)], with potential benefits in enhancing trophoblast cell functions under hypoxic conditions, inhibiting Drp1 expression by regulating miR-34a-5p, and slowing mitochondrial fission (Zhou J. et al., 2022). However, the pharmacological effects of drugs often manifest through complex network interactions rather than linear relationships. For instance, quercetin potentially mitigates miscarriage onset through a multi-target, multi-pathway mechanism. Here, in contrast to previously published studies, we use network pharmacology to identify novel mechanisms of action and targets by which quercetin ameliorates RSA and validate these by molecular docking and cellular experiments. These findings provide fresh perspectives and scientific evidence regarding the ability of quercetin to ameliorate RSA.

## 2 Materials and methods

### 2.1 Screening of quercetin-related targets

**FIGURE 1:**
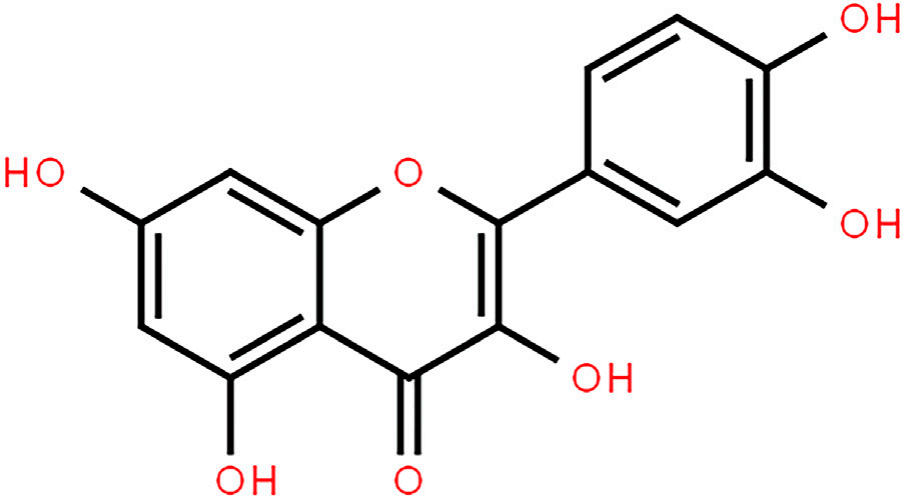
Chemical structure of quercetin.

Downloads were made from the Traditional Chinese Medicine Systems Pharmacology Database and Analysis Platform (TCMSP; https://old.tcmsp-e.com/tcmsp.php) for quercetin-related targets and annotated using UniProt. The chemical structure of quercetin was also downloaded from the TCMSP database, together with a query for the ADME (absorption, distribution, metabolism, and excretion) parameters (Figure 1; Table 1) including DL, OB, Caco-2, BBB, and the “Lipinski rule of five” (MW, AlogP, TPSA, Hdon, and Hacc).

**TABLE 1:** Pharmacological effects of quercetin.

MW	AlogP	Hdon	Hacc	OB (%)	Caco-2	BBB	DL	FASA-	TPSA	RBN	HL
302.25	1.50	5	7	46.43	0.05	−0.77	0.28	0.38	131.36	1	14.40

### 2.2 Screening for disease-related targets

The Comparative Toxicogenomics Database (CTD) (http://ctd base.org/) was searched for disease targets associated with RSA using the search term “abortion habitual”.

### 2.3 Protein–protein interaction (PPI) network construction

Venn diagrams (https://bioinformatics.psb.ugent.be/webtools/Venn/) were used to obtain targets of quercetin associated with RSA. The STRING database (https://cn.string-db.org/) was then used to construct a PPI network of these targets, which was, visualized with Cytoscape 3.9.1. The top ten key targets were screened using the degree algorithm with the cytoHubba plug-in.

### 2.4 Enrichment analysis

Gene ontology (GO) and Kyoto Encyclopedia of Genes and Genomes (KEGG) analyses were conducted to elucidate the functions of the screened target genes. Enrichment analyses were carried out using the DAVID and Reactome databases, with the threshold for statistical significance set at *P* < 0.05. Results were graphically depicted using bubble charts via the Microbiome platform (https://www.bioinformatics.com.cn/).

### 2.5 Molecular docking

The mol2 file for quercetin was obtained from the TCMSP database, hydrogenated, and converted to a pdbqt file using AutoDock. The pdb format files of the key targets were downloaded from the Protein Data Bank, dehydrated, deallocated, hydrogenated with PyMOL and AutoDock, and converted into pdpqt files. Finally, molecular docking of quercetin and key targets was performed using AutoDock, and the results were visualized using PyMOL.

### 2.6 Cell culture

HTR-8/SVneo cells (obtained from the China Center for Type Culture Collection, Wuhan, China) were cultured in RPMI 1640 medium containing 1% penicillin and 10% fetal bovine serum (Gibco, Thermo Fisher Scientific, United States) in 5% CO_2_ at 37°C in an incubator (Thermo Fisher Scientific, United States).

### 2.7 Preparation of quercetin for cell treatment

Powdered quercetin (CAS no. 117-39-5, Beijing Solarbio Science, China) was dissolved in dimethyl sulfoxide to give a 10 mM stock solution and stored at −20°C. For the experiments, quercetin was further diluted with RPMI 1640 medium to final quercetin concentrations of 1 μM, 5 μM, and 10 μM.

### 2.8 CCK-8 assay

Cells in the logarithmic growth phase were harvested and inoculated at a density of 5 × 10^5^ cells/mL at 100 μL per well in 96-well plates, and cultured in an incubator (5% CO_2_, 37°C) for 24 h. Then, the culture medium was replaced with 2% fetal bovine serum (FBS) RPMI 1640 medium, followed by further culture for 12 h. The cells were then treated with lipopolysaccharide (LPS) and quercetin. In all experimental groups, cells were supplemented with 200 ng/mL LPS and cultured in RPMI 1640 culture medium with 10% FBS; in the quercetin treatment group, different concentrations of quercetin were added so that the final concentrations of quercetin per well were 1, 5 and 10 μM, respectively. For the control group, an equal volume of medium was added instead. In this way, control, LPS, LPS + 1 μM quercetin, LPS + 5 μM quercetin, and LPS + 10 μM quercetin groups were established. Then, CCK-8 reagent (Servicebio, Wuhan, China) was mixed with RPMI 1640 medium at a ratio of 1:9, and 100 μL of the mixed solution was added to each well, followed by incubation at 37°C for 2 h. Absorbance values were measured at 450 nm.

### 2.9 Wound healing assay

Cells in the logarithmic growth phase were inoculated in six-well plates at a density of 3 × 10^5^ cells/well and cultured in 5% CO_2_ at 37°C, then, the culture medium was replaced with 2% FBS RPMI 1640 medium, followed by further culture for 12 h. When the cell density reached approximately 90%, scratches were made vertically with a 200-μL spiking gun, and cell debris was washed away with phosphate-buffered saline (PBS). Subsequently, 200 ng/mL LPS was added to cells in each experimental group, and different concentrations of quercetin were added to each well in the quercetin treatment group to give final concentrations of 1, 5, and 10 μM. The cells were photographed using an inverted microscope (Nikon, Tokyo, Japan) at 0 h and 24 h, respectively, and the width of wound healing was calculated using ImageJ software.

### 2.10 Transwell assay

For migration assessments, HTR-8/SVneo cells were adjusted to a cell density of 1 × 10^5^ cells/mL in LPS and quercetin culture medium. Subsequently, 200 μL of cell suspension was added to the upper chamber of a transwell apparatus (8 μm, Corning, United States), and 600 μL medium containing 10% FBS was added to the lower chamber. After 24 h of incubation, the cells were fixed with 4% paraformaldehyde for 30 min and stained with 0.1% crystal violet for 10 min, and images were captured under an inverted microscope. The number of cells passing through the chambers was counted using ImageJ. For the invasion experiment, the upper chamber was coated with Matrigel (1:8, BD Biosciences, United States) for 3 h before the experiment; the remaining steps were the same as those of the migration experiment.

### 2.11 Flow cytometry

HTR-8/SVneo cells were washed three times with PBS, trypsinized with EDTA-free trypsin, and centrifuged at 500 *g*, 4°C, for 5 min. Following resuspension in 1 × binding buffer, the cells were stained with Annexin V-FITC and PI solution (BD Biosciences, United States). Flow cytometry analysis was carried out using a FACScan instrument (BD Biosciences, United States). The percentages of early and late apoptotic cells were analyzed using FlowJo software (Version 10.8.1, OR, United States) to calculate the apoptosis rate.

### 2.12 RNA extraction and reverse-transcription quantitative PCR (RT-qPCR)

Total RNA was extracted using TRIzol reagent (Sangon Biotech, Shanghai, China). The concentration and purity of RNA were determined using a NanoDrop1000 (Thermo Fisher Scientific, United States), and the RNA was reverse transcribed to cDNA according to the instructions provided with the reverse transcription kit. Subsequently, PCR amplification was performed, with GAPDH serving as the internal reference gene. Primers were designed and synthesized by Shanghai Sangon Biological Engineering Co. For more details, please refer to Table 2. The RT-qPCR reaction procedure was as follows: 95°C for 30 s as an initial denaturation step, 95°C for 15 s, 60°C for 30 s, 40 cycles. The relative expression of the target genes was quantified using the 2^−ΔΔCT^ method.

**TABLE 2:** Primer sequences.

Primers	Sequences (5′–3′)
TNF-α-F	CCT​CTC​TCT​AAT​CAG​CCC​TCT​G
TNF-α-R	GAG​GAC​CTG​GGA​GTA​GAT​GAG
IL-1β-F	GCC​AGT​GAA​ATG​ATG​GCT​TAT​T
IL-1β-R	AGG​AGC​ACT​TCA​TCT​GTT​TAG​G
IL-6-F	ACT​CAC​CTC​TTC​AGA​ACG​AAT​TG
IL-6-R	CCA​TCT​TTG​GAA​GGT​TCA​GGT​TG
AKT1-F	ACT​GTC​ATC​GAA​CGC​ACC​TTC​C
AKT1-R	TCT​CCT​CCT​CCT​CCT​GCT​TCT​TG
MMP9-F	CGA​ACT​TTG​ACA​GCG​ACA​AGA​AG
MMP9-R	CGG​CAC​TGA​GGA​ATG​ATC​TAA​GC
GAPDH-F	CAC​CCA​CTC​CTC​CAC​CTT​TGA​C
GAPDH-R	GTC​CAC​CAC​CCT​GTT​GCT​GTA​G

### 2.13 Western blotting

Cells from the experimental groups were lysed with RIPA buffer containing phenylmethylsulfonyl fluoride (Solarbio Science, Beijing, China), placed on ice for 30 min, and centrifuged at 12000 *g*, 4°C, for 10 min. Protein concentration was determined using a BCA protein quantification kit. Aliquots of protein samples were separated using 12% sodium dodecyl sulfate polyacrylamide gel electrophoresis and then transferred to a polyvinylidene fluoride membrane (Millipore, United States). The membrane was blocked with 5% skim milk at room temperature, and anti-GAPDH (1:1,000; GB15004; MedChem Express), anti-AKT1 (1:1,000; HY-P74421; MedChem Express), anti-MMP9 (1:500; HY-P80425; MedChem Express), anti-caspase-3 (1:2,000; 82202-1-RR, Proteintech Group, China), anti-Bax (1:2,000; 50599-2-Ig; Proteintech Group, China), and anti-Bcl-2 (1:2,000; 12789-1-AP; Proteintech Group, China) antibodies were added, followed by incubation at 4°C overnight. HRP-conjugated secondary antibodies (1:5,000; GB23303; Servicebio) were added, followed by a further incubation for 2 h. The membranes were washed three times with Tris-buffered saline with 0.1% Tween 20 and then developed for exposure. The grey value of each protein was calculated using ImageJ, and the relative expression of each protein was calculated using GAPDH as an internal reference.

### 2.14 Statistical analysis

All experiments were repeated at least three times. GraphPad Prism 8.0 software (GraphPad Software Inc., La Jolla, CA, United States) was used for statistical analysis and graph generation. All data are expressed as the mean ± standard deviation (SD). Student’s t-test was used to measure differences between two independent groups, and one-way analysis of variance was used for multiple comparisons. The threshold for a statistically significant difference was set at *P* < 0.05.

## 3 Results

### 3.1 Screening of active components

**FIGURE 2:**
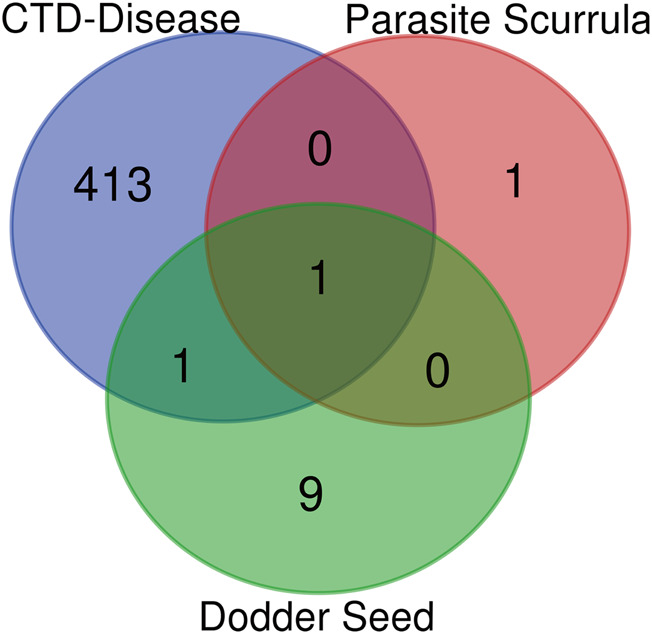
Wayne diagram of RSA and related active components of dodder seed and parasite scurrula.

In the current study, active components of Semen Cuscutae and Herba Taxilli in the kidney tonic and fetus-restoring traditional Chinese medicine Shoutai Pill were screened using the TCMSP database. The criteria of oral bioavailability ≥30% and drug-likeness ≥0.18 were used to identify 11 active ingredients of Semen Cuscutae and two active ingredients of Herba Taxilli. Detailed information on these components is presented in Table 3. Furthermore, disease-related compounds were sourced from the CTD using the keywords “habitual abortion.” Venn diagram analysis showed that quercetin is both the active ingredient in Semen Cuscutae, Herba Taxilli, and an active ingredient associated with RSA (Figure 2).

**TABLE 3:** Basic information on 12 potential active components in dodder seed and parasite scurrula.

Number	Mol ID	Molecule name	OB (%)	DL	Molecular structure
1	MOL001558	Sesamin	56.55	0.83	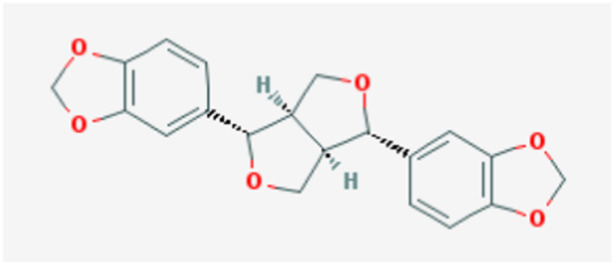
2	MOL000184	NSC63551	39.25	0.76	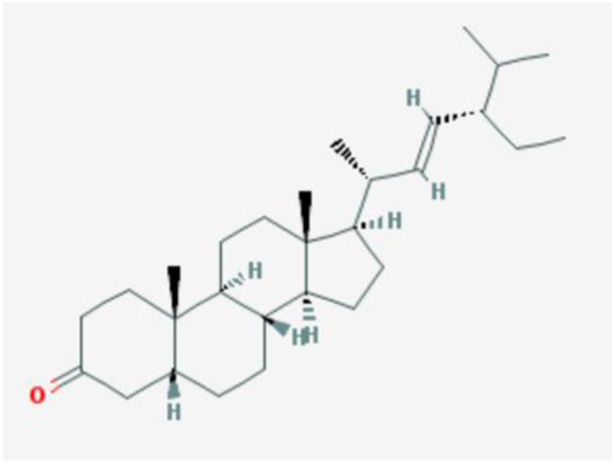
3	MOL000354	Isorhamnetin	49.6	0.31	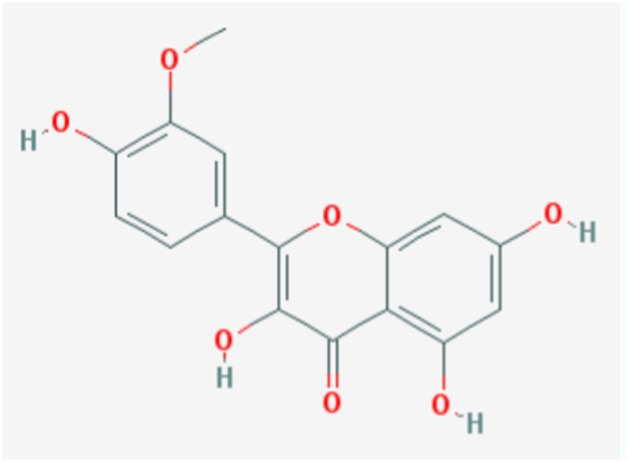
4	MOL000358	Beta-sitosterol	36.91	0.75	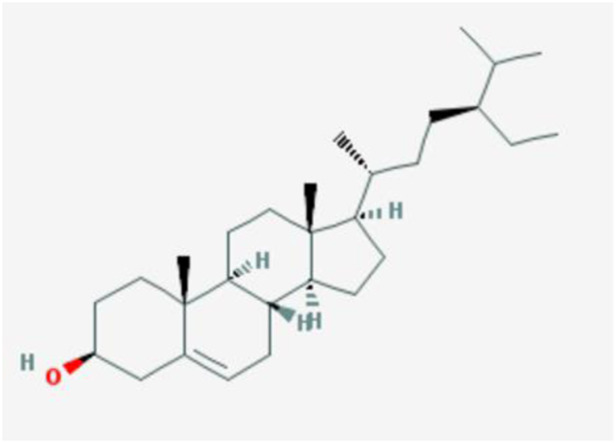
5	MOL000422	Kaempferol	41.88	0.24	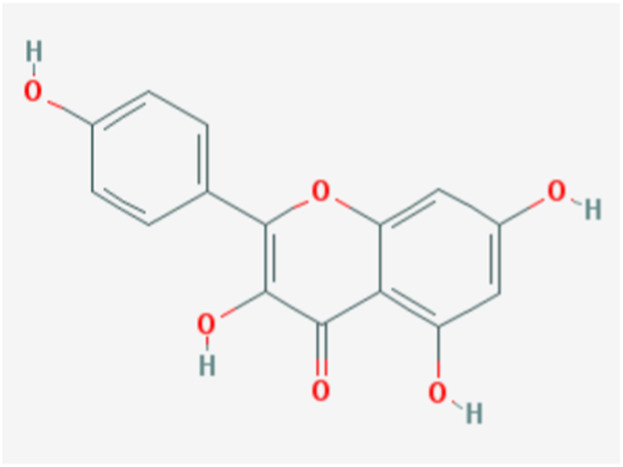
6	MOL005043	Campest-5-en-3beta-ol	37.58	0.71	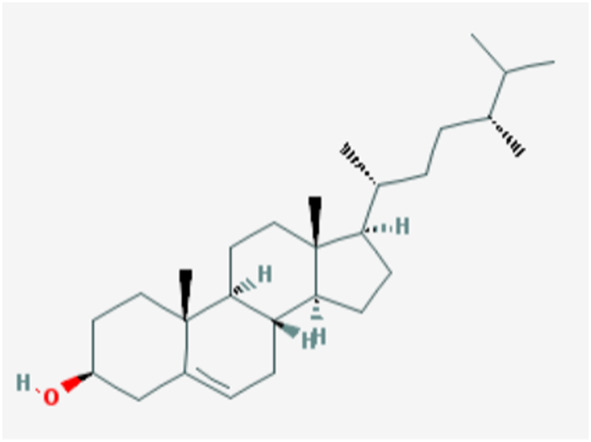
7	MOL005440	Isofucosterol	43.78	0.76	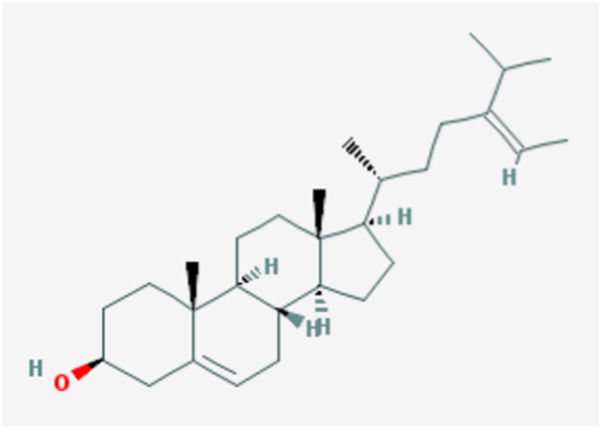
8	MOL005944	Matrine	63.77	0.25	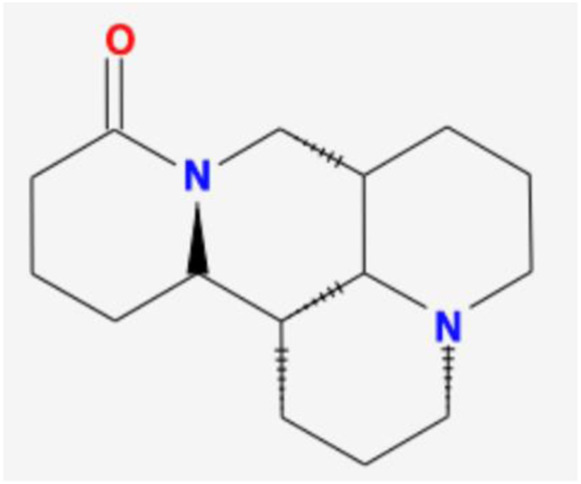
9	MOL006649	Sophranol	55.42	0.28	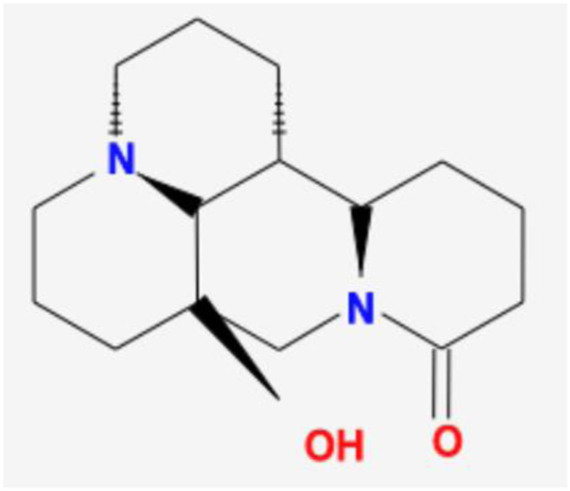
10	MOL000953	CLR	37.87	0.68	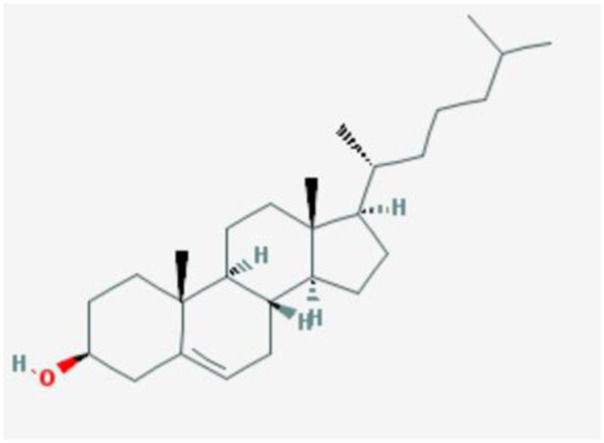
11	MOL000098	Quercetin	46.43	0.28	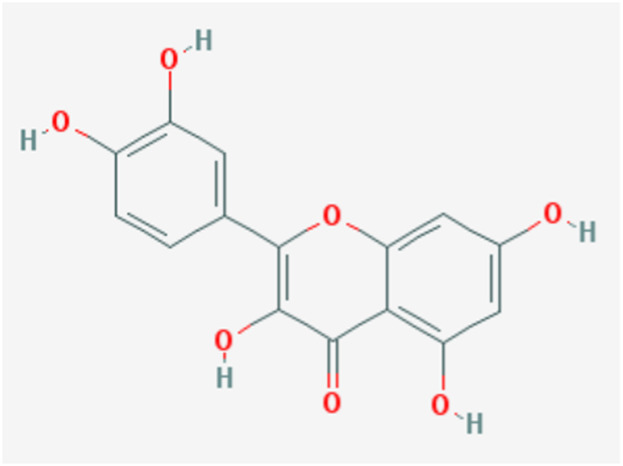
12	MOL000359	Sitosterol	36.91	0.75	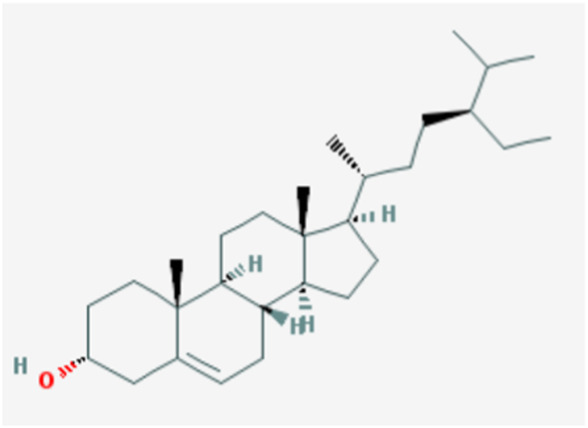

### 3.2 Screening for common gene targets of quercetin and RSA

**FIGURE 3:**
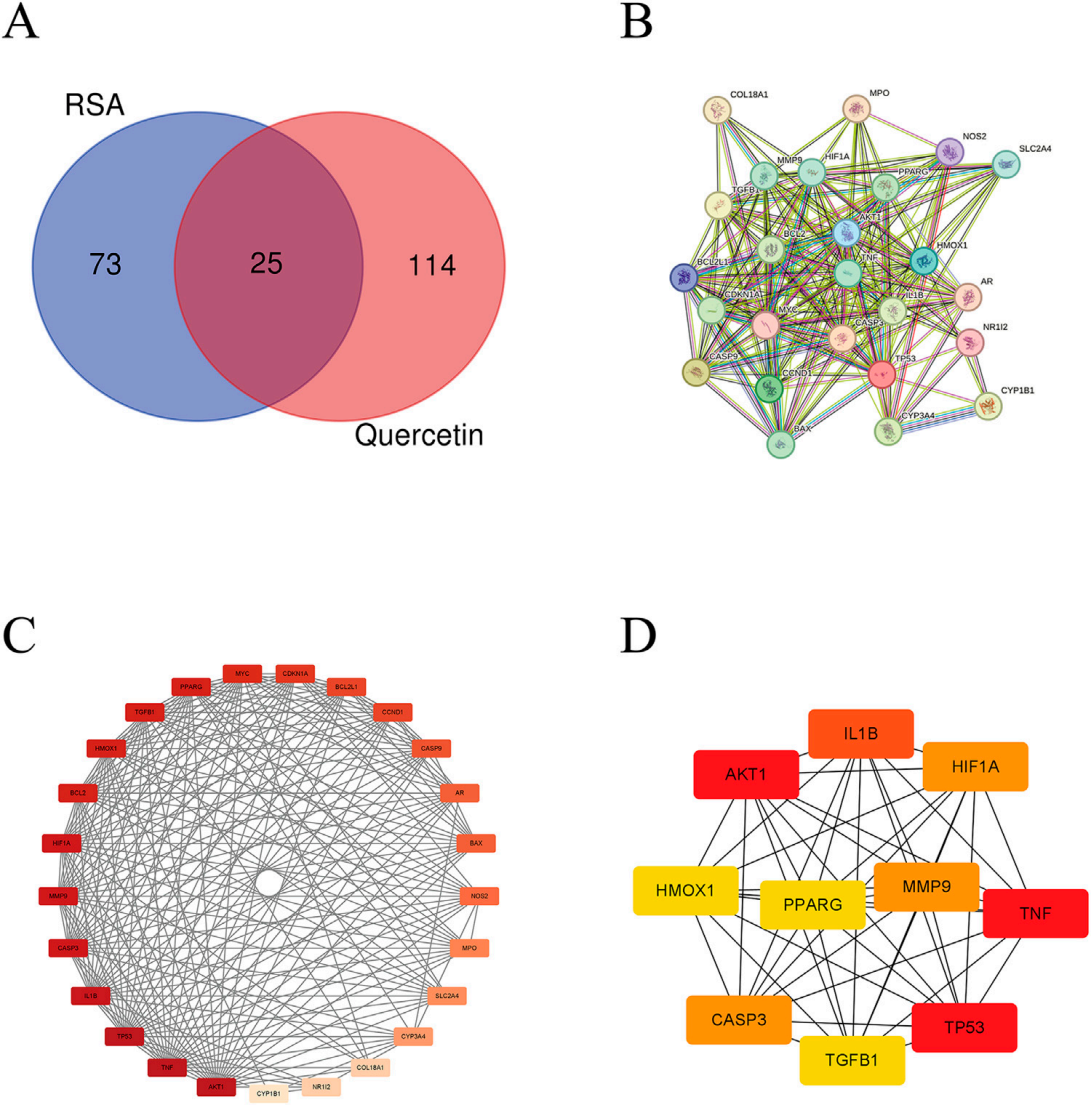
Construction of PPI interaction network and screening of hub genes. **(A)** Venn diagram of RSA disease targets and quercetin-associated targets; **(B)** Protein-protein interaction network mapping of 25 potential targets of quercetin for amelioration of RSA; **(C)** Intensity maps of interaction networks of potential targets for quercetin to improve RSA; **(D)** Quercetin improves screening of hub targets for RSA. The darker the color, the higher the degree.

We retrieved quercetin-related gene targets from the TCMSP database, followed by UniProt database annotation, resulting in 139 quercetin-related target genes. Concurrently, RSA-related genes were sourced from the CTD, resulting in the identification of 98 RSA-related genes. Upon intersection of these datasets, 25 common genes were identified (Figure 3A), namely, NR1I2, BCL2, TP53, HMOX1, NOS2, TNF, VEGFA, TGFB1, CASP9, MYC, CDKN1A, BCL2L1, MPO, AR, AKT1, PPARG, BAX, HIF1A, SLC2A4, CYP3A4, CYP1B1, IL1B, CASP3, CCND1, and MMP9.

### 3.3 PPI network construction

The common target genes were integrated into the STRING platform to establish a comprehensive PPI network featuring 25 nodes and 207 edges. Subsequently, the network diagram was imported into Cytoscape for network diagram analysis and image processing to obtain basic information about the network. In the diagram, the redder the node color, the higher the degree of connectivity (Figures 3B, C). The top ten key genes in the PPI network were screened by degree value using the CytoHubba plugin, including key targets such as AKT1, TP53, TNF, IL-1β, MMP9, and CASP3 (Figure 3D).

### 3.4 GO functional enrichment and KEGG pathway enrichment analysis of overlapping target genes

**FIGURE 4:**
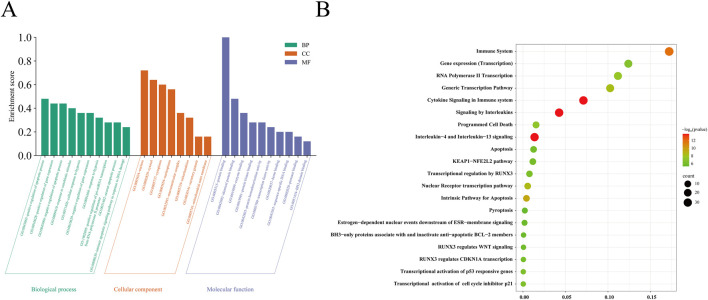
GO enrichment and KEGG pathway enrichment analysis of the 25 potential therapeutic targets. **(A)** GO enrichment analysis: *P*< 0.05; **(B)** Top 20 KEGG pathway enrichment analyses: The larger the circle, the greater the number of corresponding target genes in the term. The redder the color, the more meaningful the *P* -value.

The 25 common target genes were subjected to GO functional and KEGG pathway enrichment analyses using the DAVID and Reactome databases. A total of 340 biological processes, 29 cellular components, and 47 molecular functions were identified. The biological processes were mainly involved in positive regulation of the apoptotic process, cellular response to hypoxia, and apoptotic signaling pathway in response to DNA damage. The main cellular components were the macromolecular complex, nucleus, nucleoplasm, secretory granule, and mitochondrion; and the molecular functions were mainly associated with enzyme binding, protein binding, protein kinase binding, and transcription factor activity (Figure 4A). KEGG pathway-enriched molecules were involved in a total of 412 pathways, mainly related to interleukin-4 and interleukin-13 signaling, cytokine signaling in the immune system, apoptosis, RNA polymerase II transcription, programmed cell death, KEAP1–NFE2L2 signaling, RUNX3 regulation of WNT signaling, and pyroptosis (Figure 4B).

### 3.5 Establishment of a quercetin–target–pathway network

**FIGURE 5:**
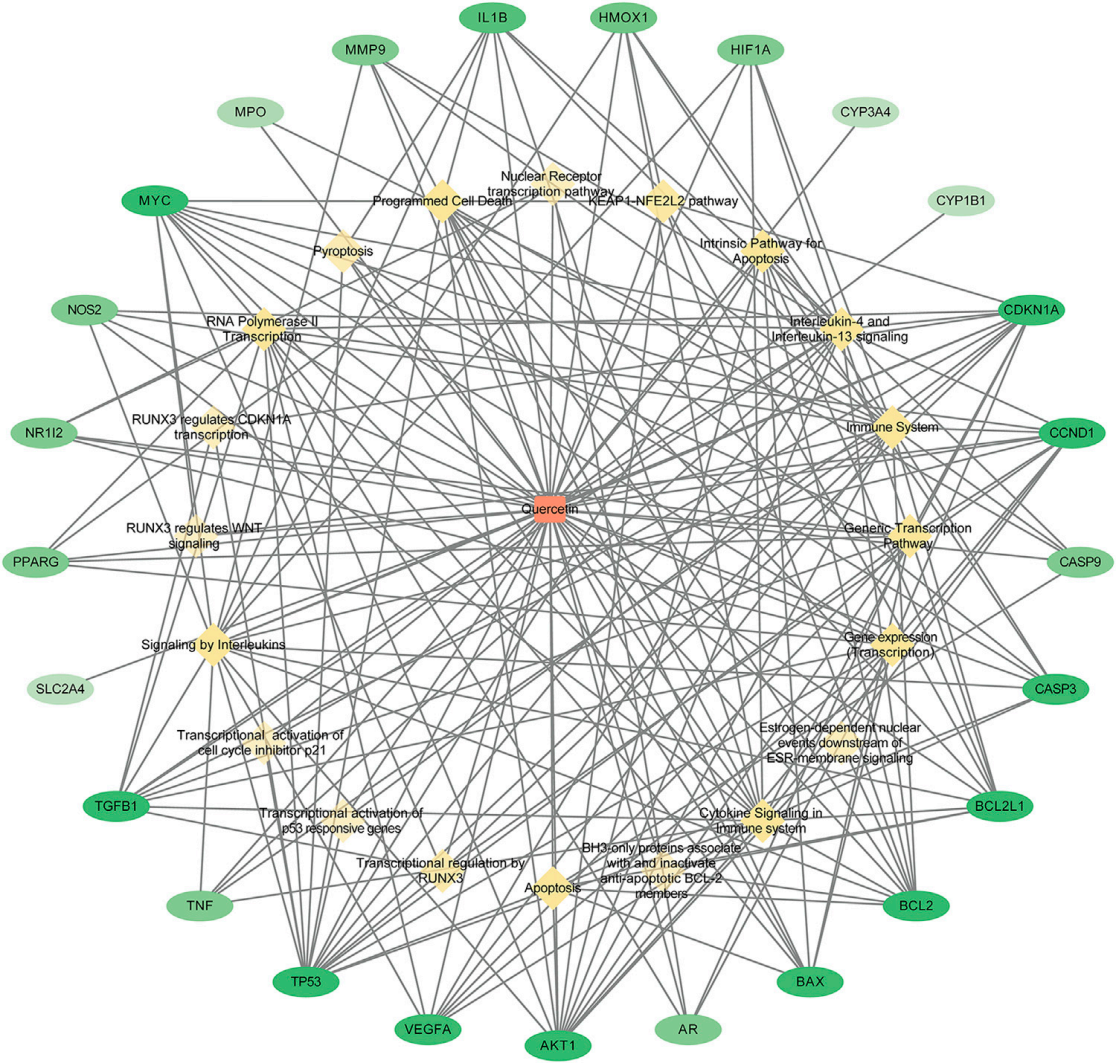
Quercetin - target - pathway network diagram. (Red squares represent quercetin, yellow diamonds represent pathways and green ovals represent gene targets; the larger the node degree value, the darker the color in the graph).

We constructed quercetin–target–pathway networks based on KEGG pathway enrichment analysis using Cytoscape 3.8.0 to discover key biological mechanisms. The network included 46 nodes and 197 bar edges associated with means by which quercetin could improve RSA; in the diagram, red squares represent quercetin, yellow diamonds represent pathways, and green ovals represent gene targets, with larger node degree values indicated by darker colors (Figure 5). The average degree of freedom was 8.565, and there were eight target genes with degrees greater than 8.565: TP53 (degree = 16), AKT1 (degree = 13), CDKN1A (degree = 13), BCL2 (degree = 11), MYC (degree = 11), CCND1 (degree = 11), TGFB1 (degree = 10), and BCL2L1 (degree = 10). The results suggested that quercetin may regulate cytokine signaling in the immune system, interleukin signaling, programmed cell death, and apoptosis via these targets to reduce the occurrence and development of RSA.

### 3.6 Molecular docking

**FIGURE 6:**
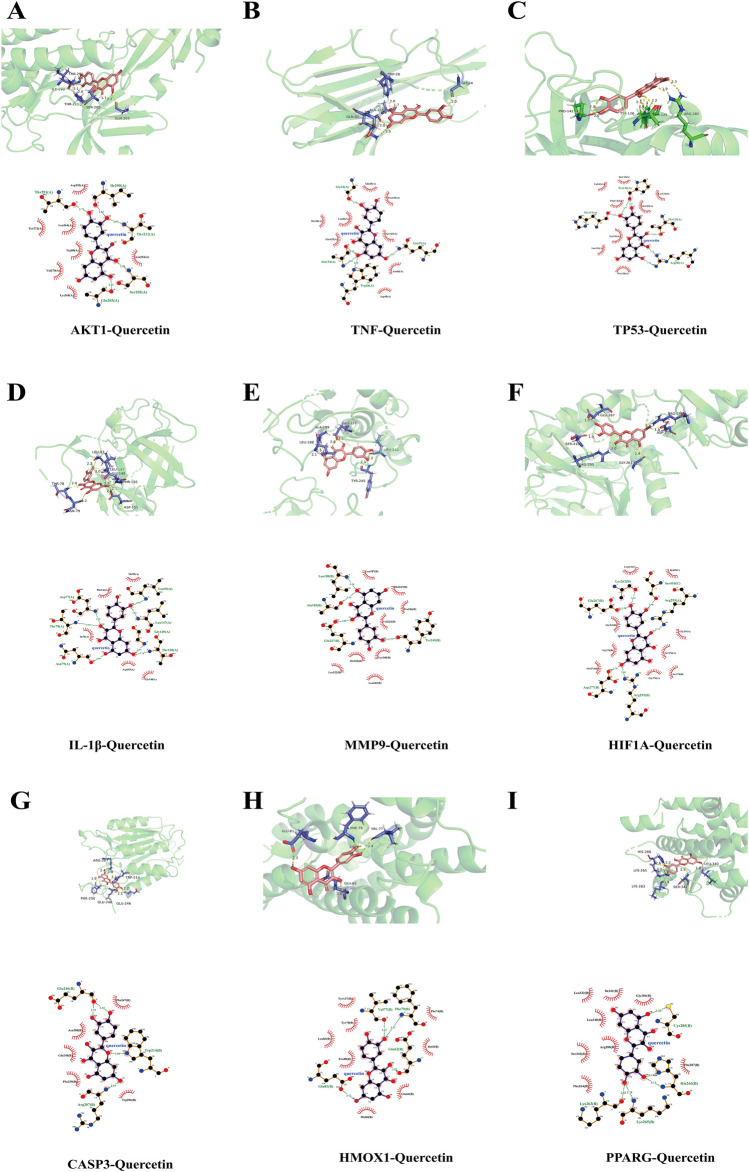
Visualisation of molecular docking 3-dimensional and 2-dimensional diagrams **(A)** AKT1- Quercetin **(B)** TNF-Quercetin **(C)** TP53-Quercetin **(D)** IL-1β- Quercetin **(E)** MMP9- Quercetin **(F)** HIF1A- Quercetin **(G)** CASP3- Quercetin **(H)** HMOX1- Quercetin **(I)** PPARG- Quercetin. Note: Blue rods are protein molecules; Pink rods are ligand molecule; Dotted lines are the hydrogen bonds.

Based on the PPI network, ten key target genes were selected for molecular docking with quercetin. The docking results are shown in Table 4 (lower scores indicate higher affinity). Quercetin has the strongest and most stable binding to MMP9, followed by AKT1, PPARG, IL-1β, HIF1A, HMOX1, CASP3, TNF, and TP53, all with binding energies less than −5 kcal/mol. Three-dimensional and two-dimensional visualizations of specific docking sites of quercetin and target proteins were performed using PyMOL and LigPlus software (Figures 6A–I). Binding of the compounds to the target proteins was dominated by hydrogen bonding and hydrophobic interactions. Quercetin formed hydrogen bonds with MMP9 at the Leu-188, Ala-189, and Glu-227 sites, and interacted hydrophobically with residues Leu-187, Met-247, Pro-246, Val-223, Tyr-248, His-226, Leu-222, and Leu-243. Similarly, quercetin formed hydrogen bonds with AKT1 at the Ile-290, Thr-221, Ser-205, and Gln-203 sites and hydrophobically interacted with residues Asp-292, Asn-204, Lys-268, Val-270, Trp-80, Leu-264, and Tyr-272. Other target proteins also formed hydrogen bonds and hydrophobic interactions with quercetin.

**TABLE 4:** Docking results of quercetin with key target molecules.

Active ingredient	Proteins	PDBID	Binding energy/(kcal/mol)
Quercetin	AKT1	4ejn	−7.53
	TNF	5uui	−6.42
	TP53	4mzi	−6.37
	IL-1β	3pok	−7.21
	MMP9	4wzv	−9.15
	HIF1A	5l9v	−7.12
	TGFB1	5vqp	−4.71
	HMOX1	1ozl	−7.1
	PPARG	2vsr	−7.46
	CASP3	3kjf	−6.86

### 3.7 Effect of different LPS concentrations on HTR-8/SVneo cell viability

**FIGURE 7:**
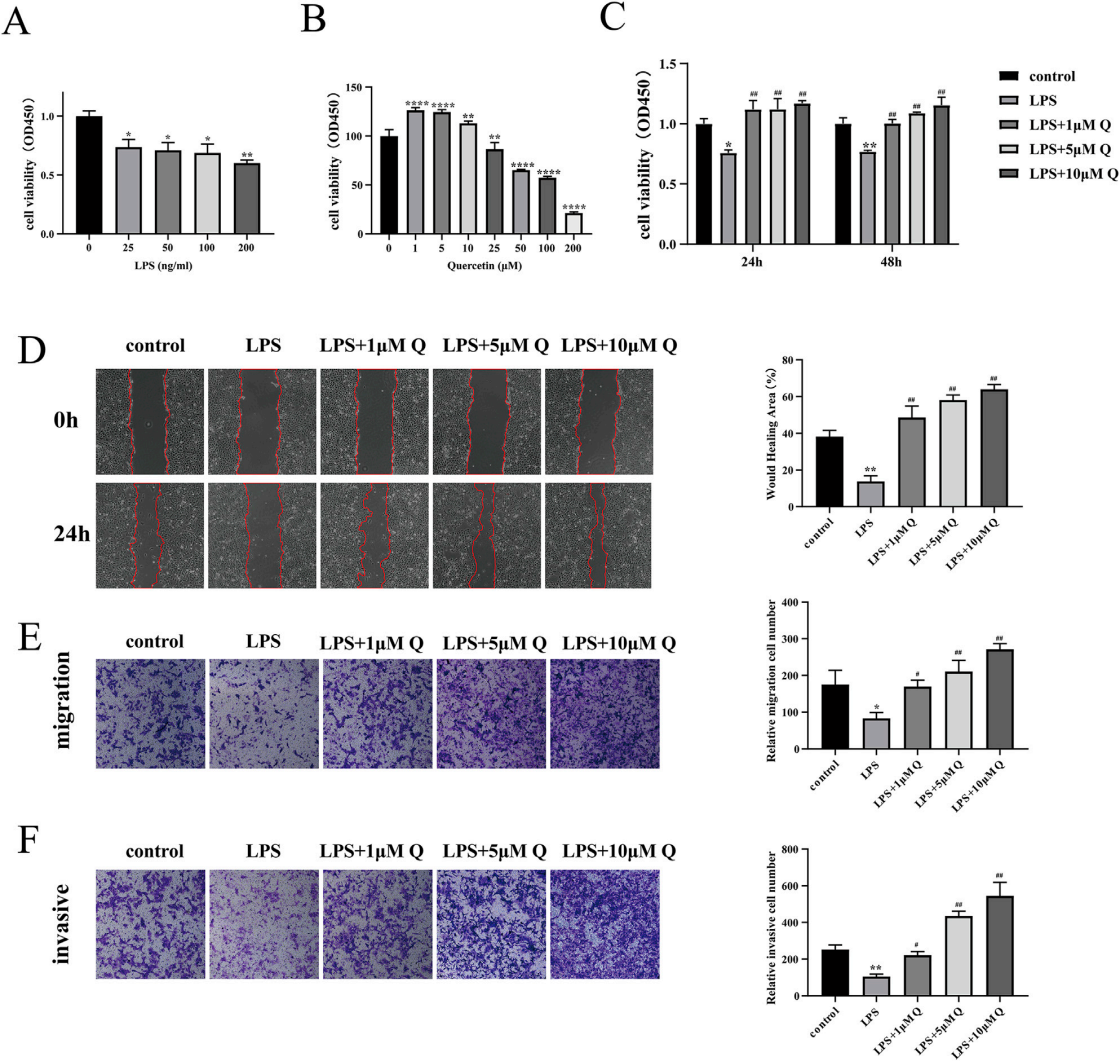
Effect of quercetin on proliferation, migration and invasion of LPS-treated HTR-8/SVneo cells. **(A)** HTR-8/SVneo cells were treated with different concentrations of LPS for 24 h and cell viability was determined by the CCK-8 assay. **(B)** Different concentrations of quercetin were applied to HTR-8/SVneo cells for 24 h and cell viability was assessed using the CCK-8 assay. **(C)** Quercetin (1, 5, and 10 μM) was applied to LPS-treated HTR-8/SVneo cells for 24 and 48 h and cell viability was assessed using the CCK-8 assay. **(D)** Cell migration was determined using the wound healing test at 0 h and 24 h. **(E,F)** The Transwell assay detects the migration and invasion ability of cells and invades cells in the lower chamber for calculation. Data are presented as mean ± SD. *P < 0.05, **P < 0.01 compared with the control group. ^#^ *P* < 0.05, ^##^ *P* < 0.01 compared with the LPS group.

HTR-8/SVneo cells were induced with 0 ng/mL, 25 ng/mL, 50 ng/mL, 100 ng/mL, and 200 ng/mL LPS, and the activity of the cells was determined after 24 h of culture. Notably, compared with the LPS group, the 25 ng/mL, 50 ng/mL, 100 ng/mL, and 200 ng/mL LPS groups showed significantly compromised cell activity, and the differences were statistically significant, with the most pronounced effect observed at 200 ng/mL (*P* < 0.01) (Figure 7A). Based on the effects of different concentrations of LPS on cell viability, shown in Figure 7A, the most appropriate concentration of LPS for treatment of HTR-8/SVneo cells was considered to be 200 ng/mL. Consequently, 200 ng/mL LPS was selected to induce inflammation in HTR-8/SVneo cells in subsequent experiments.

### 3.8 Effects of quercetin on proliferation, migration, and invasion of LPS-Treated HTR-8/SVneo cells

In order to investigate the effect of quercetin on trophoblast cells, different concentrations of quercetin were attempted to be applied to HTR-8/SVneo cells, and the viability of the cells was detected after 24 h. The results showed that 1 μM, 5 μM, and 10 μM quercetin significantly promoted cell viability, but showed an inhibitory effect on cell viability as the concentration increased (Figure 7B). Therefore for subsequent experiments we chose 1 μM, 5 μM, and 10 μM quercetin to treat LPS-induced HTR-8/SVneo cells. Experimental groups included control, LPS (200 ng/mL), and quercetin intervention (with quercetin concentrations of 1 μM, 5 μM, and 10 μM) groups, and the impact of different treatments on HTR-8/SVneo cell growth was examined. The results showed a significant decrease in cell activity in the LPS group compared with the control group (Figure 7C) (*P* < 0.01). Compared with the LPS group, the 1, 5, and 10 μM quercetin intervention groups showed significant promotion of HTR-8/SVneo cell growth after 24 and 48 h of stimulation (*P* < 0.01). In the wound healing experiments, the LPS group exhibited significant reduction in cell migration after 24 h compared with the control group (*P* < 0.01). Conversely, the 1, 5, and 10 μM quercetin intervention groups demonstrated substantial enhancements in the migration abilities of LPS-treated HTR-8/SVneo cells (*P* < 0.01) (Figure 7D). In the transwell assay, the number of cells crossing the chambers in the LPS group was significantly lower than that in the control group (*P* < 0.05). By contrast, the quercetin intervention groups exhibited notable increases in cell migration and invasion, underscoring the beneficial effects of quercetin in restoring cellular function post-LPS induction (Figures 7E, F).

### 3.9 Effect of quercetin on apoptosis in LPS-Treated HTR-8/SVneo cells

**FIGURE 8:**
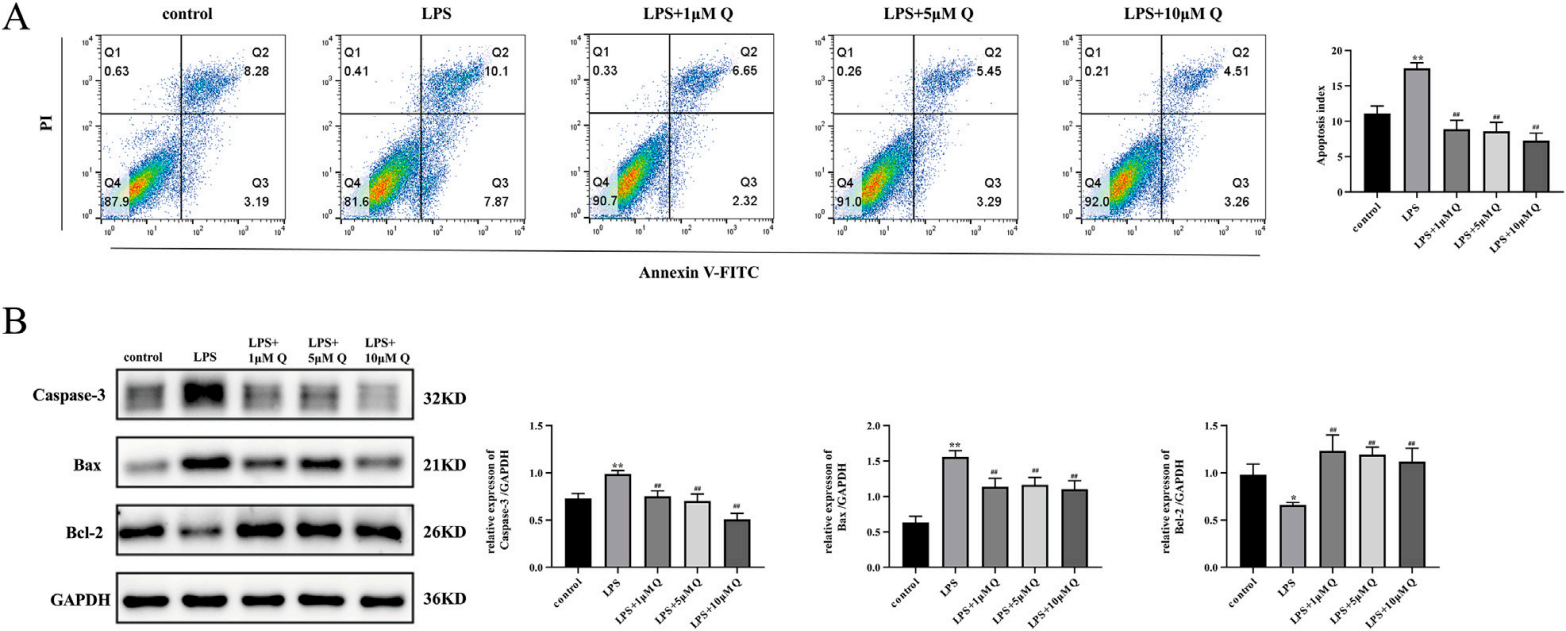
Effect of quercetin on apoptosis in LPS-treated HTR-8/SVneo cells. **(A)** Apoptosis of LPS-treated HTR-8/Sveno cells treated with 1, 5, and 10 μM quercetin were detected by flow cytometry, and the percentage of apoptosis was calculated. **(B)** Expression of apoptosis-related proteins was detected by immunoblotting. The grey scale values of each group were analyzed and the relative expression of proteins was calculated using ImageJ software. Data are presented as mean ± SD. *P < 0.05, **P < 0.01 versus the control group. ^#^ *P* < 0.05, ^##^ *P* < 0.01 compared with the LPS group. ns: no significant differences compared with the LPS group.

We used bioinformatics analyses to predict the involvement of quercetin in various apoptosis-related pathways. We also tested experimentally whether quercetin treatment could change the apoptosis of cells after LPS induction using Annexin V-FITC/PI staining and flow cytometry. The results indicated a significantly higher percentage of apoptotic cells in the LPS group compared with the control group (*P* < 0.05). Notably, the 1, 5, and 10 μM quercetin intervention groups exhibited marked reductions in apoptosis rates compared with the LPS group (Figure 8A). Western blotting analyses showed that apoptosis was promoted in the LPS group compared with the control group, resulting in increased expression levels of Bax and Caspase-3 proteins and decreased expression of Bcl-2 protein. By contrast, 1, 5, and 10 μM quercetin attenuated the onset of apoptosis, and protein levels in these groups showed the opposite results, with statistically significant differences (Figure 8B).

### 3.10 Quercetin decreased the levels of pro-inflammatory cytokines in LPS-Treated HTR-8/SVneo cells

**FIGURE 9:**
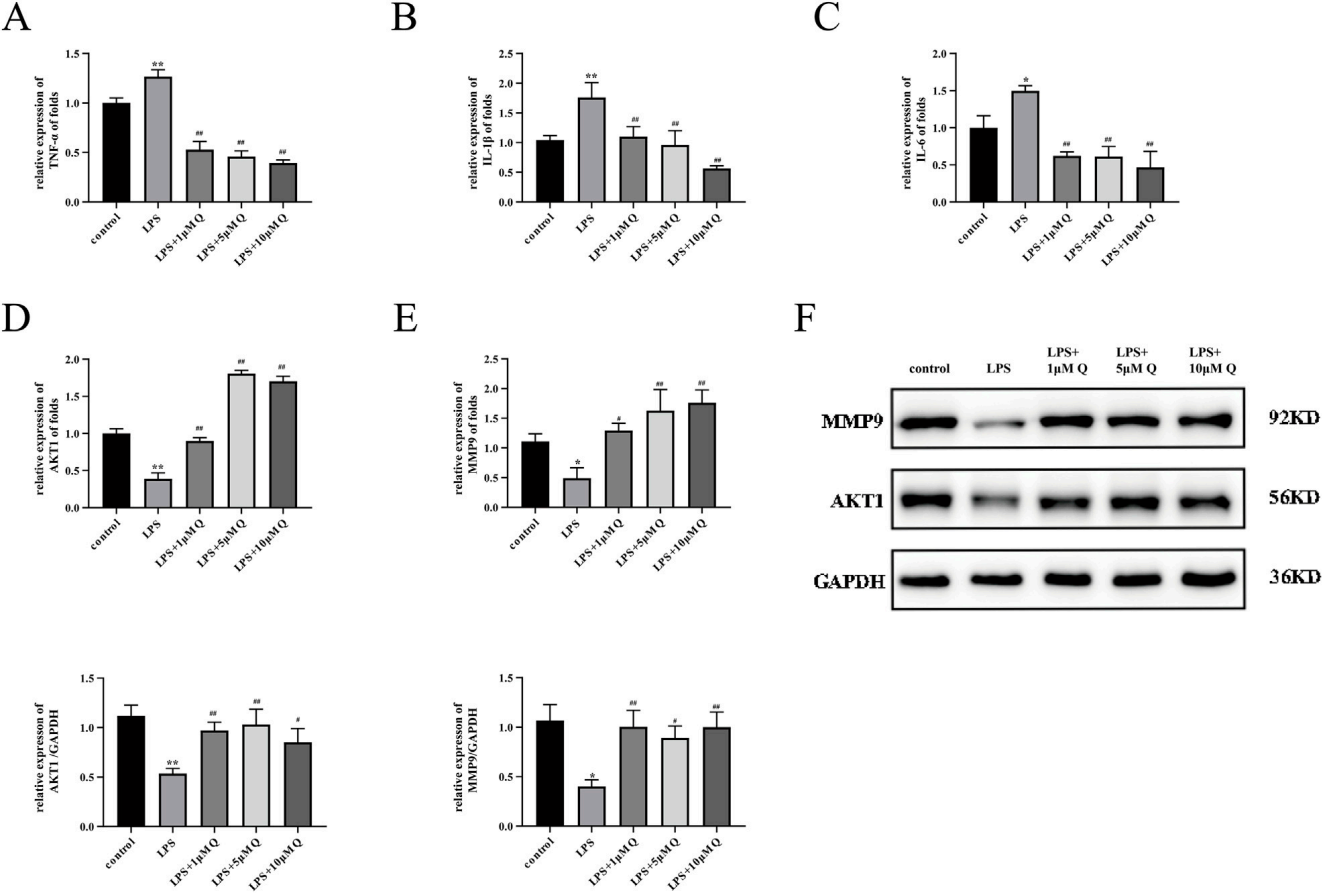
Effect of quercetin on expression of inflammatory factors and AKT1 and MMP9 proteins in LPS-treated HTR-8/SVneo cells. **(A–E)** Expression of TNF-α, IL-1β, IL-6, AKT1, and MMP9 mRNA by RT-qPCR after 24 h of LPS-treated trophoblast cells with 1, 5, and 10 μM quercetin. **(F)** Expression of AKT1 and MMP9 proteins was detected by immunoblotting after 48 h of LPS-treated trophoblast cells with 1, 5, and 10 μM quercetin. Data are presented as mean ± SD. *P < 0.05, **P < 0.01 compared with the control group. ^#^ *P* < 0.05, ^##^ *P* < 0.01 compared with the LPS group.

The inflammatory response at the maternal–fetal interface is the final pathological outcome in many RSA patients (Zhang et al., 2023). Molecular docking results indicate that quercetin has good binding energy with TNF-α and IL-1β. We detected the expression of TNF-α, IL-6, and IL-1β mRNA in the different treatment groups by RT-qPCR. The results showed that LPS significantly increased the expression of TNF-α, IL-6, and IL-1β mRNA. Compared with the LPS group, the 1, 5, and 10 μM quercetin intervention groups showed decreased expression of TNF-α, IL-6, and IL-1β mRNA (Figures 9A–C).

### 3.11 Quercetin increased levels of AKT1 and MMP9 in LPS-Treated HTR-8/SVneo cells

Molecular docking analyses underscored promising binding interactions of quercetin with AKT1 and MMP9. Subsequent experimental results confirmed the ability of quercetin to enhance the expression of AKT1 and MMP9 mRNA and protein, supporting its potential to ameliorate RSA (Figures 9D–F).

## 4 Discussion

RSA, which is classified in the category of “miscarriage” in Traditional Chinese Medicine, is characterized by “repeated pregnancies ending in abortion.” The pathogenesis of RSA is attributed to “kidney qi insufficiency and qi and blood deficiency,” as mentioned in the Collected Essentials of Women’s Science: “A woman’s kidney is connected to the fetus, which is the mother’s true qi and the child’s dependency. If the kidney qi is deficient, it will not be able to consolidate the fetal elements” (Sun et al., 2022). Therefore, Chinese medicine treats this disease by “tonifying the kidney, strengthening the spleen, promoting qi, and nourishing the blood” (Zhu et al., 2014). The Shoutai Pill, a prevalent Chinese medicinal formula renowned for its efficacy in fortifying the kidneys, enhancing spleen function, optimizing qi flow, and stabilizing fetal development, originates from medical applications in the west recorded by the late Qing Dynasty physician, Zhang Xichun. Comprising Semen Cuscutae, Herba Taxilli, Dipsaci Radix, and Colla Corii Asini, this prescription has demonstrated substantial therapeutic benefits in the prevention and treatment of RSA and is the first choice of traditional Chinese medicine for the treatment of abortion (Zeng et al., 2020; Liang et al., 2022). We have found through network pharmacology that quercetin is one of the active constituents of Semen Cuscutae, Herba Taxilli, as well as being an active constituent in the treatment of RSA. Similarly, Zhou J. et al. (2022) developed a Chinese herbal formula BYLY for the treatment of RSA based on clinical experience; this can be used to treat patients with different patterns of RSA, and its therapeutic efficacy has been confirmed by clinical trials. They also identified quercetin as an important component within this formulation. Therefore, our study used quercetin as a starting point to explore new mechanisms of action and targets for improvement in recurrent miscarriage.

Numerous studies have shown that polyphenols, including quercetin, oleuropein, resveratrol, hypericin, and ruerarin, have positive health-promoting effects on the human body (Karioti and Bilia, 2010; Martiniakova et al., 2022; Wang et al., 2022). Studies have also shown that quercetin has a variety of pharmacological effects. Supplementation with quercetin in early pregnancy increases the body’s iron stores, reduces oxidative-stress-induced DNA damage, and improves the ratio of total cholesterol to high-density lipoprotein cholesterol (Vanhees et al., 2011; Takashima et al., 2021). In animal studies, quercetin has been shown to modulate the immune microenvironment at the maternal–fetal interface, leading to a decrease in the ratio of CD4^+^/CD8^+^ T cells and IFN-γ/IL-4 in the decidua tissue of LPS-induced pregnant mice, which resulted in a decreased rate of embryo resorption and reduced incidence of miscarriage (Wang et al., 2011). Similarly, Lin et al. (2020) showed that quercetin improved preterm birth rates and offspring survival in LPS-induced mice by inhibiting the NF-κB/AP-1 pathway. Quercetin can act as an antioxidant; it has also been shown to remove senescent decidual cells and promote decidualization of endometrial stromal cells, which in turn facilitates successful embryo implantation (Kusama et al., 2021). In addition, Quercetin increases levels of GSH, reduces oxidative stress, and promotes the invasion of trophoblasts in cells with HR-induced HTR-8/SVneo (Ebegboni et al., 2019). Yoshida et al. also showed that quercetin inhibits the production of reactive oxygen species, maintains mitochondrial function, and promotes the fusion of trophoblast cells (Yoshida et al., 2024). Other polyphenolic compounds including oleuropein and resveratrol were found to significantly increase expression levels of MMP-2 and MMP-9 in HTR-8/SVneo cells and promote cell migration and invasion (Zou et al., 2019; Pirković et al., 2023). Furthermore, HTR-8/SVneo cells treated with hypericin could promote proliferation, migration, and invasion through the JAK1/STAT3 pathway (Mu et al., 2023). Some polyphenolic compounds are capable of exerting anti-inflammatory and apoptosis-inhibiting effects in trophoblast cells. For instance, puerarin was found to reverse the inhibitory effect of TNF-α on trophoblast cell viability, reduce the production of inflammatory factors, and inhibit apoptosis (Guo et al., 2022). Similarly, another experiment showed that puerarin could block H_2_O_2_-induced apoptosis and growth inhibition of HTR-8/SVneo cells by regulating upregulation of the VEGFA/Akt signaling pathway (He et al., 2022). However, resveratrol inhibited the occurrence of apoptosis by reducing the expression of IL-1β and caspase-1 through antioxidant and autophagy regulation (Li M. et al., 2020).

Using the TCMSP database, we identified 139 targets associated with quercetin. Further screening using CTD identified 25 disease targets associated with quercetin that could potentially be used to improve RSA. By filtering by degree scores, we identified potential core targets, including MMP9, CASP3, TP53, AKT1, IL-1β, and TNF. MMP9 has been found to be closely associated with migration and invasion. High levels of MMP9 prevent degradation of the endometrial extracellular matrix and inhibit intercellular tight junctions, thereby providing favorable conditions for extravillous trophoblast invasion (Jing et al., 2023). Qi et al. (2022) found that quercetin enhanced the migration and invasion of HTR-8/SVneo cells by upregulating MMP9 expression. Furthermore, strong experimental evidence shows that caspase-3, TP53, and AKT1 are closely linked to trophoblast apoptosis or proliferation. The delicate balance between trophoblast proliferation and apoptosis underpins successful pregnancies. Disruption of this equilibrium, with predominance of apoptosis among trophoblast cells, is a significant contributor to miscarriages in RSA (Atia, 2017; Chen et al., 2022). Overexpression of miR-371a-5p triggers the apoptosis pathway in trophoblast cells, markedly elevating caspase-3 protein levels (Du et al., 2019). In a related context, activation of the TP53/caspase-3 apoptotic pathway by lnc-HZ01 exacerbates the incidence of miscarriage (Huang et al., 2022). AKT1 plays a key part in the regulation of various processes, including cell growth, differentiation, and inhibition of apoptosis, and AKT1-deficient mice display increased rates of embryonic mortality and cardiac anomalies (Chang et al., 2010). TNF-α and IL-1β are pro-inflammatory cytokines that have been shown to have a variety of immune-modulatory roles during different phases of human reproduction. Previous studies have also found high expression of IL-1β and TNF-α in decidua tissue and blood in patients with RSA (Zhang et al., 2016; Löb et al., 2021), and the use of TNF inhibitors can be used to treat patients with RSA and improve live birth rates (Wu et al., 2021). These findings suggest that quercetin could potentially ameliorate the development of RSA through modulation of these genes.

Our KEGG enrichment analysis showed that the therapeutic effects of quercetin on RSA primarily involve signaling pathways associated with immune response, programmed cell death, and apoptosis. Abnormal immune responses lead to secretion of various pro-inflammatory cytokines. Within the physiological milieu of the maternal–fetal interface, the balance of Th1/Th2 cytokines skews towards Th2 dominance, which is crucial for successful embryo implantation, placental maturation, and fetal sustenance (Wang et al., 2020; Yang et al., 2022). Some studies have reported high levels of pro-inflammatory cytokines and chemokines, including IL-6, IL-8, IL-18, and TNF-α, in the blood and decidua of RSA patients. This upsurge in pro-inflammatory mediators adversely affects the migration and invasion capabilities of normal trophoblast cells, posing challenges for successful embryo implantation (Zavatta et al., 2023). By contrast, bacterial LPS, a key constituent of Gram-negative bacterial cell walls, modulates inflammatory cascades by inducing various inflammatory factors (Page et al., 2022). A study found that administering 0.1 μg of LPS to mice in early pregnancy resulted in 100% embryo loss rate by day 7 of gestation. High levels of Th1-type cytokine IFN-γ have been found in LPS-induced abortive mice, whereas levels of Th2-type cytokine IL-4 were significantly reduced (Zhong et al., 2002; Wang et al., 2011). Many studies have shown that an imbalance in apoptosis disrupts fetal development and leads to miscarriage. Apoptosis is prevalent in the placental villous tissue where inflammation occurs, and placental inflammation is capable of inducing cell death (Deftereou et al., 2012). The development of RSA is closely linked to apoptosis and inflammatory responses, implying a potential causal link between innate immune activation triggered by LPS in trophoblast cells and persistent pregnancy-specific inflammation in RSA.

The HTR-8/SVneo cell line serves as a pivotal model in contemporary trophoblast function research. In the present study, *in vitro* experiments were used to elucidate the mechanism of action of quercetin in ameliorating RSA and validate the results of network pharmacological analyses. The results showed that 200 ng/mL of LPS markedly suppressed HTR-8/SVneo cell activity, diminishing cell migration and invasion, inducing apoptosis, and upregulating expression of inflammatory factors IL-1β, TNF-α, and IL-6, confirming the successful establishment of an LPS-induced inflammatory model. Treatment with 1, 5, and 10 μM quercetin significantly enhanced cell viability, promoted migration and invasion, curbed apoptosis, and reduced pro-inflammatory cytokine concentrations in LPS-induced HTR-8/SVneo cells. Molecular docking analyses revealed favorable binding energies of quercetin with MMP9 and AKT1, with values of −9.15 and −7.5 kcal/mol, respectively. Our experimental results also showed that MMP9 and AKT1 mRNA and protein expression levels were lower in the LPS group compared with the control group. However, quercetin treatment promoted the expression of these mRNAs and proteins, providing evidence at the cellular level to support the effectiveness of quercetin in ameliorating RSA.

## 5 Conclusion

New targets and mechanisms of action by which quercetin may help to prevent abortion have been identified using network pharmacology. The network pharmacological results highlight key targets including AKT1, TP53, TNF, IL-1β, MMP9, and CASP3 that could have roles in the treatment of RSA. These targets have been shown to ameliorate the onset and development of RSA through cytokine signaling in the immune system, interleukin signaling, programmed cell death pathway, apoptosis, etc. The molecular docking results provide further evidence that AKT1 and MMP9 bind strongly to quercetin. In addition, cellular experiments provide reliable data supporting the ability of quercetin to exert anti-inflammatory effects, inhibit apoptosis, and ameliorate the inflammatory response in both mother and fetus by upregulating AKT1 and MMP9 and downregulating IL-1β, TNF-α, and IL-6. Consequently, quercetin may be an effective drug for the treatment of RSA. The results presented here also open up potential new research opportunities for natural compounds in the treatment of RSA. However, this study had some limitations, and further evaluation of the effectiveness of quercetin through *in vivo* experiments will be needed in the future.

## Data Availability

Publicly available datasets were analyzed in this study. This data can be found here: https://old.tcmsp-e.com/molecule.php?qn=98, https://ctdbase.org/detail.go?type=disease&acc=MESH%3AD000026.

## Abbreviations

RSA, Recurrent spontaneous abortions; CTD, Comparative Toxicogenomics Database; TCMSP, Traditional Chinese Medicine Systems Pharmacology Database and Analysis Platform; PPI, Protein–protein interaction; GO, Gene ontology; KEGG, Kyoto Encyclopedia of Genes Genomics; RT-qPCR, Reverse transcription-quantitative polymerase chain reaction; TBST, Tris-buffered saline containing 0.1% Tween-20; PBS, Phosphate Buffered Saline; FBS, Fetal bovine serum; CCK-8, Cell counting kit-8; LPS, Lipopolysaccharide; MMP9, Matrix metallopeptidase 9; AKT1, AKT serine/threonine kinase 1; CDKN1A, Cyclin dependent kinase inhibitor 1A; MYC, MYC proto-oncogene; CCND1, Cyclin D1; Bax, BCL2 associated X; BCL2, BCL2 apoptosis regulator; TP53, Tumor protein p53; TNF, Tumor necrosis factor; IL-1β, Interleukin 1 beta; IL-6, Interleukin 6; HR, Hypoxia reoxygenation.
